# Enterprise Security for the Internet of Things (IoT): Lightweight Bootstrapping with EAP-NOOB

**DOI:** 10.3390/s20216101

**Published:** 2020-10-27

**Authors:** Aleksi Peltonen, Eduardo Inglés, Sampsa Latvala, Dan Garcia-Carrillo, Mohit Sethi, Tuomas Aura

**Affiliations:** 1 Department of Computer Science, Aalto University, 02150 Espoo, Finland; sampsa.latvala@aalto.fi (S.L.); mohit.sethi@aalto.fi (M.S.); tuomas.aura@aalto.fi (T.A.); 2 Department Information and Communication Engineering (DIIC), Faculty of Computer Science, University of Murcia, 30100 Murcia, Spain; eduardo.ingles@um.es; 3 Odin Solutions (OdinS), 30820 Murcia, Spain; dgarcia@odins.es; 4 NomadicLab, Ericsson Research, 02420 Kirkkonummi, Finland

**Keywords:** EAP-NOOB, contiki, IoT, bootstrapping, security

## Abstract

The emergence of radio technologies, such as Zigbee, Z-Wave, and Bluetooth Mesh, has transformed simple physical devices into smart objects that can understand and react to their environment. Devices, such as light bulbs, door locks, and window blinds, can now be connected to, and remotely controlled from, the Internet. Given the resource-constrained nature of many of these devices, they have typically relied on the use of universal global shared secrets for the initial bootstrapping and commissioning phase. Such a scheme has obvious security weaknesses and it also creates undesirable walled-gardens where devices of one ecosystem do not inter-operate with the other. In this paper, we investigate whether the standard Extensible Authentication Protocol (EAP) framework can be used for secure bootstrapping of resource-constrained devices. EAP naturally provides the benefits of per-device individual credentials, straightforward revocation, and isolation of devices. In particular, we look at the Nimble out-of-band authentication for EAP (EAP-NOOB) as a candidate EAP authentication method. EAP-NOOB greatly simplifies deployment of such devices as it does not require them to be pre-provisioned with credentials of any sort. Based on our implementation experience on off-the-shelf hardware, we demonstrate that lightweight EAP-NOOB is indeed a way forward to securely bootstrap such devices.

## 1. Introduction

The Internet of Things (IoT) promises to integrate a large number of physical devices, such as light bulbs, electronic locks, and various household appliances, into networks of connected smart objects. Connecting these devices to the Internet allows for remote access and control without the need for any direct physical interaction.

Many large home appliances, such as smart TVs and refrigerators, typically reuse the existing Wi-Fi infrastructure for Internet connectivity because of its low marginal cost for connecting any number of devices. However, at the same time, there is a growing number of IoT devices that rely on different low-power radio technologies, such as Zigbee [1], Thread [2], and Z-Wave [3]. These devices typically form a mesh network and must rely on a gateway or a hub for Internet connectivity. An example of such devices are the Internet-connected light bulbs from Philips [4], Ikea [5] and Osram [6]. While smart light bulbs are a popular example, a plethora of other devices, including switches, power sockets, and window blinds, also rely on radio technologies other than the traditional Wi-Fi network.

The reasons for vendors of these devices to use radio technologies other than the widely deployed Wi-Fi network can include extended range, lower cost, lower energy requirements, and support for mesh networking. Regardless of the radio technology used by these smart devices, their owners must provide the necessary security and configuration parameters before they can start using their devices. Security, usability, and scalability of this initial bootstrapping phase is critical. A security breach at this early stage in the lifecycle of a smart device will naturally leave it vulnerable to attacks later on. Similarly, requiring complex user interactions would deter their widespread adoption.

IoT devices currently use a very wide variety of techniques for this initial security bootstrapping phase. Sethi [7] categorizes the available bootstrapping techniques and lists some methods that are used by existing devices as well as standard protocols. The bootstrapping techniques not only significantly vary in their implementations and manufacturer requirements, but also expect users to perform substantially different tasks. Hence, there is a need for standard, secure, and inter-operable bootstrapping solutions that work with a wide range devices in many different deployment settings without requiring major changes to the tasks that a user must perform.

In this paper, we specifically look at secure bootstrapping of devices, such as smart light bulbs, which may form a mesh network and connect to the Internet through a hub. We investigate whether the Extensible Authentication Protocol (EAP) framework [8], which is widely used for bootstrapping enterprise Wi-Fi devices, is also suitable for resource-constrained devices that use other radio technologies with slightly different deployment setups. Our main contributions are as follows:

(1) We chronicle a concise but illustrative overview of existing bootstrapping protocols designed for resource-constrained devices. Our overview describes protocols developed in research literature and standards while also examining the bootstrapping processes of real-world off-the-shelf devices.
(2) We identify important design principles for bootstrapping protocols and present arguments in favor of a standards-based bootstrapping solution that is based on the EAP framework.
(3) We provide an overview of the EAP-NOOB protocol along with its security properties and implement it on Zolertia Firefly breakout boards.
(4) The EAP-NOOB protocol implementation is complemented with the implementation of a novel out-of-band channel that works with a simple small LED light and a mid-range smartphone camera. This OOB channel can accommodate the limitations of a resource-constrained platform and still achieve data transfer rates of 80 bps.
(5) We present an initial evaluation of the memory, time, and message size requirements of our bootstrapping solution.

The rest of the paper is structured, as follows. Section 2 discusses the relevant state of the art in secure bootstrapping of resource-constrained devices. In Section 3, Section 4 and Section 5, we discuss why EAP is a suitable choice for bootstrapping resource-constrained IoT devices. We also provide an overview of the EAP-NOOB protocol and its security properties that make it a desirable authentication method. Section 6 presents the operating system that we chose for our implementation and discusses its significant features. We explain our implementation setup in Section 7 and evaluate it in Section 8. Finally, Section 9 discusses the significance of our findings and Section 10 concludes the paper.

## 2. Background

We begin this section by studying the standard specifications of existing popular low-power radio technologies and documenting their bootstrapping methods. Thereafter, we discuss the user experience of the bootstrapping process for common off-the-shelf devices. Finally, we look at some secure bootstrapping proposals that have been suggested in the research literature.

### 2.1. Standards

Zigbee [9] is an IEEE 802.15.4-based open standard specified by the Zigbee alliance for creating and maintaining short-range personal area networks (PANs). It is intended for low-cost appliances, such as those used in home automation and health care. Over the years, various application profiles for the Zigbee standard have been developed, each defining the message formats and processing requirements for complying devices [10]. For example, the Zigbee Light Link (ZLL) [11] profile is specified for controlling lights in consumer households, while the Zigbee Home Automation (ZHA) [12] profile is specified for smart home appliances, such as wireless window shades and intruder alarms.

The bootstrapping method used by Zigbee devices varies between the different application profiles. For example, the ZLL profile uses a master key for deriving the active network key when a new device joins an existing network. This master key must be known by all devices implementing the protocol and it is provisioned at the time of the device manufacturing. Therefore, ZLL implicitly assumes that all devices (and manufacturers) implement sufficient security for storing and processing the master key. However, in 2015, the master key of the ZLL profile was leaked out on Twitter, compromising the security of all previously manufactured devices while using the protocol [13]. Similarly, in the Zigbee Home Automation profile, a default link key is used as a fallback method for unknown devices. An attacker can use this fallback mode to sniff traffic and decipher the active network key [10].

The Thread stack [2] is an open standard for IPv6-based low-power device-to-device communication. Similarly to Zigbee, it builds upon the IEEE 802.15.4 link layer. Thread defines protocols that are necessary for forming, joining, and maintaining networks of low-cost wireless devices that are used in home automation, lighting, and healthcare applications. The security of Thread networks is primarily based on a network-wide shared passphrase [14]. This passphrase can be a well-known weak default, such as `123456’ used by Nordic Semiconductor Thread devices [15], or it can be configured by the user. A well-known weak default passphrase obviously makes the entire network vulnerable. User configured network-wide passphrases are naturally better than well-known defaults. Thread relies on the Password-Authenticated Key Exchange by Juggling (J-PAKE) protocol for preventing dictionary attacks against user-selected passphrases. However, relying on the same network-wide passphrase still makes the system fragile, as compromise of a single device can leak the shared passphrase of the entire network. Additionally, using the same passphrase across all devices renders revocation of individual devices extremely challenging: a user would have to configure the new passphrase on all of the remaining devices in the network.

Z-Wave [3] is a proprietary standard that is intended for home automation applications. It operates in the unlicensed Industrial, Scientific, and Medical (ISM) band. Z-Wave relies on a primary node: a network controller for managing all other devices in the network. Earlier versions of Z-Wave used a factory provisioned key for delivering the network key protecting the link-layer traffic. Fouladi and Ghanoun [16] claimed in 2015 that this factory provisioned key is 16 bytes of zeros. Nowadays, Z-Wave uses a new S2 security protocol [17] for bootstrapping devices. The S2 protocol requires each device to have a factory provisioned asymmetric key pair. The device also has the first 16 bytes of the 32 byte public key printed as a QR code and a 40 digit string. A new device joining an existing network advertises its public key. The key is garbled by setting the first 16 bits to zero. A user then manually adds the missing 16 digits to the network controller by scanning the QR code of the new device (or copying the first five digits of the 40 digit printed string). Thereafter, the network controller and the new device perform an Elliptic Curve Diffie-Hellman (ECDH) key exchange. Using the session key resulting from the ECDH key exchange, the network controller securely sends the network key used for protecting traffic among nodes. The development of the S2 protocol shows the natural need for having individual device identities and keys.

The Zigbee IP [1] application profile uses a significantly different bootstrapping method when compared to other standards discussed above. Authentication in the network is based on using the Extensible Authentication Protocol (EAP). In particular, Zigbee IP uses certificates with Transport Layer Security (TLS), i.e., EAP-TLS as the authentication method. The standard requires manufacturers to setup a Public Key Infrastructure (PKI) and install device certificates at the time of manufacturing. Certificates, together with EAP-TLS, allow for device authentication without any user action. Once EAP-TLS authentication is successfully completed, then the resulting keys are used to protect the configuration information sent by the controller to the new device.

Zigbee Light Link and Zigbee IP both rely on some form of manufacture provisioned credentials. While these credentials can be used for verifying that devices belong to a certain manufacturer, they provide no information about the device owner deploying them. Therefore, it is possible that a Zigbee light bulb may connect to the controller of a neighbor who also owns devices from the same manufacturer. The Zigbee IP specification realizes this potential issue of joining the wrong network. Several mechanisms for overcoming the challenge of correct network selection are mentioned in section 6.4 of the Zigbee IP specification [1]. One of the suggestions states that joining nodes could scan for beacons and discover all Zigbee IP networks in its radio range. Joining nodes would then display information about all of the networks and allow users to select the correct network which the node should join.

### 2.2. User Experience with Devices

To understand how the above standards are implemented in practice, we also looked at the user experience of bootstrapping two off-the-shelf devices. The Philips Hue [4] ecosystem uses the Zigbee Light Link (ZLL) profile. A typical Hue deployment consists of a bridge device, a smartphone application, and up to 50 connected light bulbs per bridge. The bridge acts as a centralized control system that allows for the user to administer connected light bulbs through the application. To set up a new Hue network, users need to connect the Hue bridge to the Internet through their home router. Users then pair their smartphone and the bridge device. This is done by simultaneously tapping a button on the bride and the smartphone application. The smartphone must be connected to the same home router to which the bridge is attached before initiating the pairing process. Once the pairing process is successfully completed, the bridge can be accessed through the application and used to control the light bulbs that are connected to it. New lights can be added with the application either by performing an automatic search of available devices within range of the bridge, or by manually entering the serial number of the light bulb that the user wishes to connect.

FIBARO [18] is a wireless smart home automation system based on the Z-Wave [3] protocol. Much like a Philips Hue system, it requires a centralized hub, called a Home Center, which is also controlled through a smartphone application. The FIBARO system supports a variety of devices, such as sensors, switches, buttons and dimmers, all accessible through the hub. Adding or removing new appliances to the network typically requires physical access. For example, the FIBARO Flood sensor [19] contains a hardware button used for both connecting and disconnecting it from the hub. To add or remove the sensor, it needs to be placed within range of the controller, after which the button is pressed three times. It is important to note here that physical access to devices is not only required for configuring new devices, but also for revoking network access of devices configured in the past.

### 2.3. Proposed Solutions in Research Literature

We examined existing research literature to find other bootstrapping protocols that use EAP as a building block. Slim EAPOL (SEAPOL) [20] is a lightweight variation of the standard EAP over Local Area Network (EAPOL) [21]. According to the authors, SEAPOL provides the same functionalities as EAPOL, but with significantly less overhead. Instead of the six bytes required by EAPOL, it only uses three bits. They argue that SEAPOL, therefore, is a more suitable option when bootstrapping resource-constrained devices.

Trust Extension Protocol for Authentication in Networks Oriented to Management (TEPANOM) is an authentication, bootstrapping, and configuration protocol for IoT devices, introduced by Jara [22]. Pawlowski et al. [23] implement TEPANOM over SEAPOL as a lightweight alternative for authenticating and bootstrapping resource-constrained devices. They claim that the combination is one of the most lightweight options when compared to other authentication and bootstrapping methods.

The protocol for Carrying Authentication for Network Access (PANA) [24] is a network layer protocol that transports EAP messages over User Datagram Protocol (UDP) [25] packets. Rather than defining a new authentication method, it offers a lightweight alternative for transporting EAP messages over an unreliable network. O’Flynn [26] suggests using PANA for the secure bootstrapping of resource-constrained devices. Hernández-Ramos et al. [27] propose a similar solution that extends PANA for secure bootstrapping of smart objects.

## 3. Design Principles

As is evident from our discussion in Section 2, many of the existing devices and standards currently do not use EAP methods for device authentication and bootstrapping. Instead, a common approach is to rely on a network-wide shared secret. For devices using the Thread stack, a network-wide shared secret is input by the user in the form of a passphrase. For devices using the Zigbee Light Link standard, a global shared secret is hard coded by the device manufacturer into all devices. Indeed, even larger appliances that use Wi-Fi networks rely on the Wi-Fi Protected Access 2 (WPA2)-Personal authentication mode, in which a single passphrase is provisioned to all of the devices.

The attack shown by Morgner et al. [13] on Zigbee Light Link highlights that using a single network-wide shared secret makes the deployment extremely vulnerable. A single compromised device can reveal the secret and make the entire system prone to attacks. Large scale Denial of Service (DoS) attacks that originate from IoT devices have also led to a strong desire for individually identifying and authenticating each IoT device as well as potentially isolating them from each other and the rest of the network infrastructure.

In this section, we identify and discuss four important design principles that guided our choice of a bootstrapping protocol for resource-constrained smart home and automation devices.

1. *Principle 1—Centralized AAA infrastructure:* initial home and small office networks were designed with the expectation that only a small number (3–5) of devices will be connected to each other and the Internet. Emergence of low-cost communication technologies, such as Zigbee and Bluetooth, along with embedded sensors has however increased the number of devices even in typical home and small office networks. The need for a central AAA server for monitoring attached devices is no longer exclusive to enterprise environments only. A centralized AAA server not only enables monitoring of all the connected devices, but also allows removing access of devices which are sold or infected.
2. *Principle 2—Fine-grained access control:* as the number of devices in a network increases, there is naturally a need for isolating them. As an example, the Z-Wave S2 protocol divides the Z-Wave network into three dedicated security classes (“S2 Access Control”, “S2 Authenticated”, and “S2 Unauthenticated”), each of which have a unique network key. A bootstrapping protocol must support individual device authentication, key provisioning, and the isolation of devices from each other as well as the rest of the network after the initial bootstrapping.
3. *Principle 3—Cloud-hosted service:* it is naturally unreasonable to expect average home owners to setup and maintain AAA servers. Outsourcing this functionality to the cloud will ensure better security and allow more frequent updates. A bootstrapping protocol should therefore support deployment of AAA servers on-site (for small enterprises) as well as in the cloud (for home environments).
4. *Principle 4—Flexibility of credentials:* IoT devices are developed by a diverse set of manufacturers and they vary significantly in terms of their capabilities. While some IoT devices are comparable to general purpose computing devices, others may be resource limited. A bootstrapping protocol should ideally be able to scale up or down, depending on the device capabilities while also supporting different types of credentials that are be available.

## 4. EAP for Bootstrapping

EAP relies on a central Authentication Authorization and Accounting (AAA) server infrastructure. EAP authentication results in a different Master Session Key (MSK) for each device known only to the server. The server may then send keys derived from the MSK to a gateway or hub (referred to as an authenticator in EAP terminology). Indeed, in current Wi-Fi deployments, a Pairwise Master Key (PMK) derived from the MSK is sent to the Access Point (AP). APs can then use the key to verify that the devices are authorized to join the network and send security credentials for protecting group traffic. In addition to having this deployment flexibility and per-device keys, EAP makes the revocation of devices straightforward. Unlike the process of removing a Fibaro Z-Wave sensor from the network as documented in Section 2, the owners of devices using EAP authentication do not need physical access to them for deleting their network access credentials. Owners can simply delete any device credentials on the server for removing them from the network. EAP infrastructure also provides support for devices that are mobile and may need to connect to the Internet through different gateways as they move. Finally, EAP, together with protocols, such as RADIUS [28] and DIAMETER [29], provides the flexibility of deploying the AAA server on-site or remotely.

Admittedly, the owners of devices discussed thus far cannot be expected to understand, let alone setup, the complexity of an EAP infrastructure. However, as we will see later on, the deployment can be simplified such that users only need to perform very basic tasks while adding new devices to their network. Rather than diverging from enterprise wireless deployments, EAP provides a stable standard with open source implementations that can be extended to home and small office networks. Therefore, we chose to utilize the EAP infrastructure as a critical building block for our bootstrapping protocol.

## 5. EAP-NOOB

Many different authentication methods have been specified for the EAP framework, making it suitable for building bootstrapping protocols for a variety of IoT devices. However, most of the existing EAP methods require some credentials to be pre-provisioned on the device. EAP-pwd [30] for example requires a user to input a passphrase on the device and the server. Because many IoT devices only have a limited User Interface (UI) available, this task is often non-trivial. Similarly, EAP-TLS [31] requires certificates on both the client IoT device and the AAA server. This is not only costly because of the necessity of certificates, but, as Sethi and Aura [32] argue, there is an additional cost of securing the supply chain. Moreover, servers may or may not be able to verify device certificates issued from different manufacturers (and vice-versa). This potentially allows for the creation of walled-gardens where users are locked into a single ecosystem.

Nimble out-of-band authentication for EAP (EAP-NOOB) [33] is a new EAP method currently being standardized at the IETF. EAP-NOOB allows for device authentication and bootstrapping without any pre-configured identifiers or security credentials. It not only bootstraps the device, but also binds it with a user account on the AAA server. This is an important feature for managing and re-configuring devices after they have been bootstrapped. Indeed, as we saw in Section 2, many currently deployed devices require users to have a user account through which they manage their network and devices.

EAP-NOOB is able to bootstrap devices that have no pre-configured credentials using an ephemeral Elliptic Curve Diffie–Hellman (ECDH) key exchange between the device (called the peer in EAP terminology) and the AAA server. Diffie–Hellman is convenient for bootstrapping new devices since it creates a secure association (shared secret key) between two entities without authenticating them. To prevent man-in-the-middle (MitM) attacks and to authenticate the two endpoints, EAP-NOOB relies on an user-assisted out-of-band (OOB) channel. It requires the user to deliver a single OOB message from either the peer device to the server or from the server to the peer device. This OOB message contains the critical information needed for mutual authentication [33]. The security properties of EAP-NOOB have been verified with formal security proofs [34].

As is the case with many IoT devices, EAP-NOOB specifically supports devices that have limited input/output capabilities. It supports both devices that only have an input interface (e.g., cameras), as well as those that only have an output interface (e.g., printers). However, unlike device-specific proprietary bootstrapping protocols, EAP-NOOB is an open and generic bootstrapping protocol that can support a variety of OOB channels, depending on the capabilities of the device being bootstrapped. Previous implementations [35] have, for example, used QR-codes and NFC-tags for transmitting the OOB authentication data. The EAP-NOOB specification suggests encoding the OOB message content as a Uniform Resource Locator (URL) when sending it in the from the peer device to the server [33].

The size of the message sent on the OOB channel can be in the order of tens of bytes. When encoded as a URL, the length of an OOB message is typically 70–130 characters [36]. While transferring this amount of data on an OOB channel is trivial for large appliances, such as smart TVs, it poses a significant challenge to the use of EAP-NOOB on the devices that are more resource-constrained, such as smart light bulbs.

The in-band messages of EAP-NOOB are encoded as JavaScript Object Notation (JSON) objects. EAP-NOOB relies on multiple EAP conversations, called exchanges, for establishing the necessary keys and completing the bootstrapping process. During these exchanges, the peer device and the AAA server exchange configuration parameters perform the ECDH key exchange, and ensure that the OOB message has been delivered correctly. It is important to note that users only need to transfer the OOB message once during the initial bootstrapping phase. Thereafter, the peer device and the server can refresh the session key and update their configuration parameters through a repeatable Reconnect Exchange. The Reconnect Exchange also provides optional forward-secrecy and the possibility to upgrade the cryptographic algorithms used during the key exchange and derivation.

## 6. Contiki-NG

Baccelli et al. [37] note that IoT deployments use a wide variety heterogeneous hardware. The authors highlight that developers of IoT devices can significantly benefit from having an operating system that abstracts the underlying hardware and provides features such as memory management. Consequently, integrating our bootstrapping solution in an operating system can make it easier for developers to adopt it. Therefore, we implement it on a variant of the popular Contiki OS [38].

Contiki is an open-source lightweight operating system for resource-constrained devices. It provides Internet connectivity for sensors, microcontrollers, and other low-cost devices over standard IPv4 and IPv6. Additionally, Contiki supports various modern, low-power wireless protocols, such as IPv6 over Low-Power Wireless Personal Area Networks (6LoWPAN), Routing Protocol for Low-Power and Lossy Networks (RPL), and Constrained Application Protocol (CoAP) [39].

Contiki-NG [40] is an independently developed fork of Contiki OS. It aims to improve the original codebase by only focusing on standard-based IPv6 communication, supporting modern IoT platforms, improving documentation, and modernizing the structure of the code [41]. Because Contiki OS is no longer actively maintained, we instead chose Contiki-NG for our implementation. In the next few sub-sections, we present some important characteristics of Contiki-NG.

### 6.1. Processes and Scheduling

Contiki is built on an event-driven kernel, in which all processes run on the same stack [38]. In a purely event-driven OS, a dispatcher oversees process handling with event-handlers. When an internal or external event occurs, the dispatcher hands over control of the processor to the process with the corresponding event-handler [42]. Such events can be caused by for example expired timers, incoming network packets, or user actions [43]. One of the major challenges of event-driven programming is that it can cause processes that perform intensive computations to block all other execution for an undesirable amount of time. For applications that need to perform computationally intensive tasks, Contiki supports preemptive multi-threading as an application library on top of its event-driven kernel [38].

The purely event-driven model was later refined with the introduction of the protothreads library [44]. With protothreads, each application only consists of one actual process and all sub-processes are implemented as stackless threads. Protothreads allow for application developers to reduce the number of explicit state machines they would need with traditional event-driven programming. The overhead of using protothreads as an abstraction is the amount of memory required for a pointer, which, in practice, means two or three bytes, depending on the embedded system. For example, the size of a pointer in an AVR microcontroller is three bytes and in MSP430 only two bytes [44].

Contiki-NG follows the event-driven model of the original codebase [43], but it removes support for preemptive multi-threading due to a lack of documented use cases [45]. Consequently, multitasking in applications has to be implemented with events and timers. A process in Contiki-NG is a stackless protothread [44] running on top of the kernel. As was the case in Contiki, Contiki-NG also contains an event-scheduler that is responsible for handing over control of the processor to individual processes. Since the scheduler in Contiki-NG is cooperative, each protothread is expected to restore control back to the scheduler after it has finished executing the task it was assigned. Failing to do so will block other processes and cause a deadlock situation, in which all of the processes are waiting for execution time. After returning control of the processor, a protothread typically becomes idle until the next event is dispatched to it by the scheduler [46].

### 6.2. Events and Timers

Events in Contiki-NG can be triggered by either internal or external reasons. Internal events, such as the expiration of timers, are caused within the system itself, whereas external events, such as user actions, are caused by actors outside of it. Each event is either synchronous or asynchronous. A synchronous event is created for a specific process and dispatched immediately after being created. It will typically cause the posting process to hand over control of the CPU instantly and then suspend until the receiver has completed processing the event. Asynchronous events, on the other hand, are queued up and dispatched by the kernel in an unspecified order following the round-robin principle. Unlike synchronous events, asynchronous events can also be broadcast, which causes them to be dispatched to all processes.

Besides waiting for events to trigger actions, many applications typically rely on time-based tasks, such as re-transmitting lost network packets or performing tasks at intervals. In Contiki-NG, the system time is accessed through the clock module. In addition to providing libraries for monitoring time, Contiki-NG also allows processes to delay the CPU for a number of clock ticks. Based on the clock module, Contiki-NG provides a set of timer libraries. These libraries can be used by both the OS itself and by the applications running on top of it. Besides application-specific tasks and scheduling processes, timers can also be used to, for example, wake up the system from an idle low power mode after a certain time period has elapsed [47].

Out of the five currently implemented timer libraries, two are simple clock-timers, one is used for real-time task scheduling, and the remaining two for scheduling events and function calls, respectively. The two libraries most relevant for event-driven programming are the event timer (etimer) and the real-time task scheduler (rtimer). When an etimer is initialized, it is given a time interval that specifies when it expires. Once this interval has elapsed, the timer generates an event and sends it to the process that created it. By either discarding or resetting the timer after receiving the event, processes can utilize this library for sleeping or performing tasks at intervals. The rtimer library works in a similar way, but also includes a callback function. An rtimer uses its own clock module to ensure higher clock resolution when compared to other timers. Furthermore, it uses the Interrupts Service Routine (ISR) to prevent interrupts from affecting its normal operation. It also pre-empts the scheduler before running the real-time task [47].

Because etimers use events to communicate expiration of timers, system interrupts or polled events can affect its accuracy. This sacrifice of accuracy however ensures that the normal operation of the application is not affected. On the other hand, rtimers communicate expiration through interrupts. While we obtain more precision with rtimers, we also risk interrupting the execution of a critical process.

## 7. Implementation

In this section, we document our implementation of EAP-NOOB [48] on resource-constrained IoT devices running the Contiki-NG operating system. Our implementation architecture and hardware platform were chosen to model a typical smart home environment.

### 7.1. Hardware Platform

We use Zolertia Firefly [49] motes as our IoT devices. These are resource-constrained breakout boards with limited memory and input/output capabilities. Each mote has only 32 kB of RAM and 512 kB of flash memory. However, despite their limited capabilities, they provide support for hardware accelerated elliptic-curve cryptography (ECC), specifically for the NIST P-256 (secp256r1) curve. Because we expect the cryptographic calculations of EAP-NOOB to be the most resource-intensive operation, the Firefly motes are well suited as test IoT devices.

### 7.2. Architecture

Even though the standards discussed in Section 2 all have significant differences, most of them use the same components and architectural patterns. In all of these standards, the resource-constrained IoT devices form a mesh network and rely on a controller node for connecting them to the Internet. We follow the same design pattern for our implementation. Other salient features of our implementation are explained in this section.

*MAC layer:* we use the IEEE 802.15.4 radio available on the Firefly motes. It is a popular MAC layer protocol that is used as a building block for many other standards, such as Zigbee and Thread.

*Routing protocol:* routing in our implementation is based on Routing Protocol for Low-Power and Lossy Networks (RPL) [50]. RPL networks use Destination Oriented Directed Acyclic Graphs (DODAGs) for routing packets. DODAGs contain a root node that acts as a border router between the local network and the Internet.

*Application layer protocol:* constrained Application Protocol (CoAP) [51] is a lightweight protocol for application-layer messaging. It is similar to the Hyper-Text Transfer Protocol (HTTP), but takes into consideration the specific requirements of resource-constrained devices. For example, messages in CoAP are typically exchanged asynchronously over the unreliable User Datagram Protocol (UDP) [25]. Given the lightweight nature of CoAP, we chose to use the Contiki-NG implementation of CoAP for application-layer messaging between the devices and the controller. The controller itself is not constrained and uses HTTP over TLS for interacting with web services on the Internet. Thus, it acts a proxy that translates application payloads from CoAP to HTTP and vice-versa.

*EAP lower layer:* EAP messages can be transported over any lower layer. In enterprise Wi-Fi deployments, EAP is typically carried directly over the IEEE 802.11 frames. Another approach, Protocol for Carrying Authentication for Network Access (PANA) [24], specifies how EAP can be transported over UDP in a network for access authentication. It is important to note the difference between the two approaches. Mainly, in enterprise Wi-Fi networks, the device is not assigned an IP address until it is authenticated and authorized to join the network. PANA, on the other hand, allows for the device to first obtain an IP address and then prove that it is authorized. However, it can be removed from the network (i.e. packets originating from it can be dropped) if it does not authenticate within the expected time.

Garcia-Carrillo and Marin-Lopez [52] suggest the use of CoAP as a lower layer for transporting EAP messages in resource-constrained IoT networks. They argue that this approach results in shorter messages due to the lightweight nature of CoAP and that it reduces the code footprint, since the protocol can be reused after the bootstrapping phase. Due to the fact that EAP requires the order of messages to be preserved, it cannot run over raw IP or UDP. Garcia-Carrillo and Marin-Lopez ensure ordered message delivery by utilizing Confirmable CoAP messages [51]. The authors show a significant overhead reduction when using CoAP instead of PANA. These results are further confirmed by Garcia-Carrillo et al. [53] and shown to be most significant in multi-hop networks. Hence, in our implementation setup, we use CoAP-EAP for transporting EAP messages between the IoT device and the controller.

**Figure 1:**
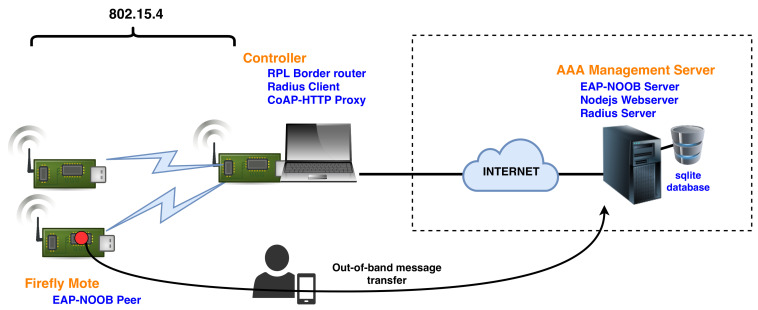
Extensible Authentication Protocol-Nimble out-of-band authentication (EAP-NOOB) deployment overview.

Figure 1 shows our overall implementation setup. Let us recall that the goal of our bootstrapping solution is to authenticate IoT devices (motes) before they are allowed to communicate with other devices in the local network and send packets to the Internet. As part of the initial authentication process, they must also be registered on a AAA management server. For this, we use a standard Ubuntu 16.10 virtual machine hosted in a private cloud. The AAA server consists of the following components:

- A web server. The web server provides a user interface for tracking IoT devices. Users are required to create an account on it before they can add or manage their devices. We use Node.js for the implementation.
- A database. We use an SQLite database for storing information about user accounts and IoT devices.
- An EAP server. We use hostapd [54] for implementing the EAP-NOOB server side components. It interacts with the web server through the shared database.
- A RADIUS server. In addition to functioning as an EAP server, hostapd also has a built-in RADIUS server for receiving RADIUS encapsulated EAP messages.

Firefly motes in our implementation are the EAP peer devices. They form an IEEE 802.15.4 mesh network and use RPL for routing. Furthermore, one of the motes is connected over USB to a laptop and serves as a controller device, a RPL border router, and an EAP authenticator. It provides Internet connectivity to all other motes after they are authenticated and ensures that new unauthenticated devices are only allowed limited connectivity, mainly to send EAP packets towards the server. As with any other typical EAP deployment, the communication between the authenticator and the EAP server must be protected. For this, we use RADIUS [28] with a shared key. Although this configuration only needs to be performed once, average users can not be expected to do it on their own. In Section 9, we will discuss how this process can be made less burdensome.

Successful EAP-NOOB authentication results in two session keys at the server and the peer—MSK and EMSK. MSK is further used to derive a third key, PMK, which is also delivered to the authenticator (controller) over RADIUS. This key is used to protect the CoAP communication between the peer device and the controller. Developers can freely choose an appropriate security protocol to use with the PMK after the secure bootstrapping phase is complete. One suitable candidate is Datagram Transport Layer Security (DTLS) [55] protocol, which is, for example, used by IKEA smart light bulbs [56].

### 7.3. OOB Communication with a Blinking LED

As is evident from the description of the EAP-NOOB protocol presented in Section 5, an out-of-band (OOB) channel is essential for bootstrapping new devices. Previous implementations of EAP-NOOB have relied on QR-codes and NFC tags [35]. Although adding small e-ink displays or NFC tags may theoretically be possible, it not only increases the cost of devices, but also significantly increases energy consumption. While investigating potential OOB channels for our test devices, we noted that they have a very small RGB LED that can be controlled programmatically. Furthermore, the EAP-NOOB specification explicitly states that it may be possible to use a blinking light for delivering the OOB message. There is plenty of research literature and past implementations of visible light communication [57,58]. However, we were faced with some unique challenges resulting from (a) a very small RGB LED, (b) an off-the-shelf mid-range smartphone, (c) a resource-constrained embedded platform, and (d) a requirement to transfer approximately 100–200 bytes within a few seconds.

**Figure 2:**
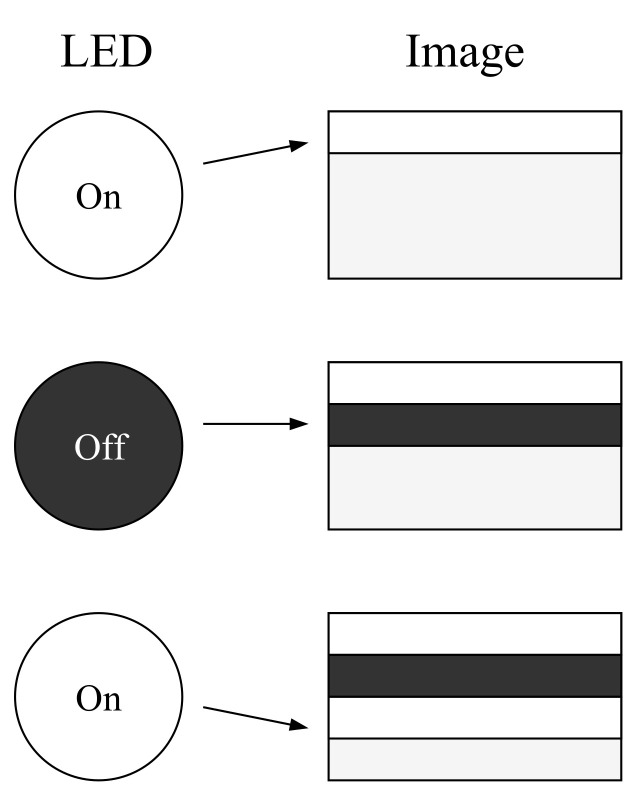
The electronic rolling shutter effect. Capturing a blinking LED leaves bright and dark bands on the image.

We now describe our implementation of an OOB channel with blinking LED lights. Modern digital active pixel sensors that are found in smartphones are typically produced using Complementary Metal-Oxide-Semiconductor (CMOS). When compared to Charge-CoupLED Device (CCD) sensors, CMOS sensors have lower power consumption and are easier to manufacture. CMOS sensor cameras capture images using an electronic rolling shutter [59]. This implies that every image is captured progressively by exposing each row of pixel sequentially. This can be utilized for transmitting data with Visible Light Communication (VLC) [60] by encoding it into a binary format and transmitting it to the camera with a blinking Light-Emitting Diode (LED). If the camera’s rolling shutter frequency is higher than the LED’s blinking frequency, dark and bright bands are captured in the image. Dark bands are captured when the LED is turned off and bright bands when the LED is turned on, as shown in Figure 2. These bands can then be mapped to represent binary data based on their luminosity, a technique that is known as On-Off keying (OOK).

**Figure 3:**
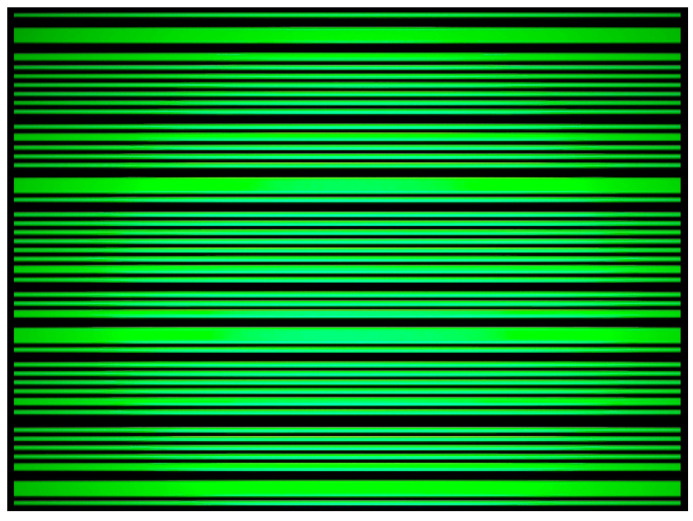
Screen capture of a blinking LED through a smartphone camera.

In our implementation, a LED acts the transmitter and a smartphone camera as the receiver. We implement the reader as an Android application for the Nokia 5.1 smartphone. The preview resolution of the camera is limited to 800×600 pixels due to the hardware limitations of the device. Therefore, the camera is only able to reliably capture 30 images per second, while simultaneously decoding each captured image in real-time. On the sender-side, we encode the OOB message with Manchester encoding for robustness and reliability. Because the whole message does not fit in one image, we split it into parts. Furthermore, to indicate the start of a data sequence we use a wider light band for a six-bit preamble (*011110*). Figure 3 shows the blinking LED seen through a smartphone camera with wider light bands indicating the preamble.

**Figure 4:**
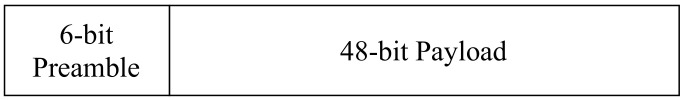
Data structure of a three-character block.

**Figure 5:**
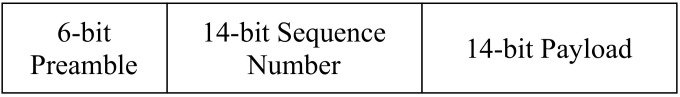
Data structure of a sequence-numbered character block.

We implement and test two basic methods for transmitting and reading data using VLC. In the first method [61], as shown in Figure 4, the preamble is followed by a 48-bit payload. We also include a prefix indicator to relay information about the length of the message to the reader. While testing transmission reliability, we noticed that the smartphone camera was not able to capture data seamlessly, causing data to be lost between captured images. We managed to solve the issue by repeating each set of three characters multiple times, which gives the camera enough time to capture all of the data successfully. However, moving the smartphone during the scanning process may disrupt the signal, requiring the process to be started over. The second method [62] utilizes sequence numbers to provide error correction. In addition, since the character set of the OOB message only consists of ASCII characters, we can use seven-bit encoding. This optimization allows for more data to be encoded within each captured image. In this variation, shown in Figure 5, the preamble is followed by a seven-bit sequence number and a seven-bit payload, both of which are Manchester encoded into 14-bit values. This means that each captured image can contain 2–3 characters.

**Figure 6:**
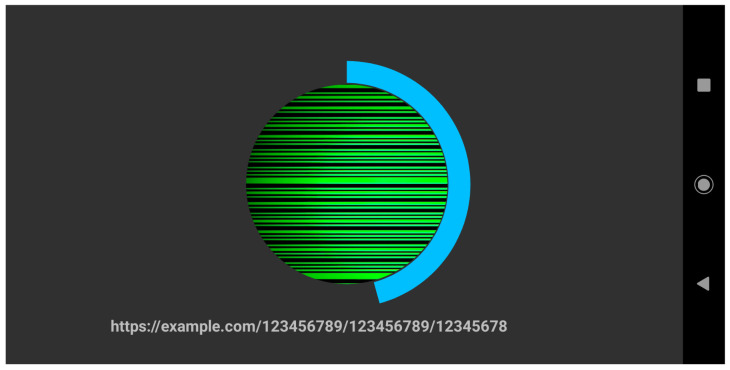
Screen capture of the decoding process. The circular loading bar shows the progress of scanned data and the text on the bottom shows the already decoded URL.

The theoretical maximum bitrate with a 800×600 resolution and a 30 FPS capture rate is approximately 18,000 bps, or 18 kbps (600 bits × 30 FPS). However, because of the way timers work in Contiki-NG, controlling the width of the band is difficult. Moreover, on and off require different timers to produce the same bandwidth. We noticed that using long bit-sequences causes some of the messages to cut short, which causes data to be lost. This is noticeable at the edges of the captured frames and causes the actual bitrate to be lower than the theoretical bitrate. Furthermore, due to the Zolertia Firefly mote’s LED being small, the camera needs to be placed directly on top of the LED to perceive the rolling-shutter effect. To help the user in scanning the small blinking LED, we implemented a circular UI design that is shown in Figure 6. Once the message is scanned, the complete URL is displayed to the user. By clicking the link, the user is taken to the website to complete the authentication process.

In EAP-NOOB, the OOB message can be encoded as a URL that consists of a domain name, a 22-byte identifier (PeerId), a 16-byte nonce (Noob), and a 16-byte hash (Hoob). With the first transmission method we were able to successfully read the message in approximately two seconds. With the second method the corresponding time was six seconds. However, the slower method based on sequence numbers is more robust against movement and scanning the OOB message can be done in parts. Conversely, while the repeating method is faster, it is more prone to errors if the user accidentally moves their hand during the scanning.

### 7.4. Bootstrapping Sequence

After being powered up, a mote starts the bootstrapping process by joining a RPL network in its vicinity. An RPL network is formed when IoT devices exchange two kinds of messages: DODAG Information Objects (DIOs) and Destination Advertisement Objects (DAOs). DIO messages contain the configuration parameters that are necessary for the devices to construct and maintain the DODAG. Once a new device joins the DODAG, it uses DAO messages to announce its parent nodes. This information is propagated to the border router at the root of the DODAG. Neither the device nor the existing RPL network have authenticated each other at this point. Hence, they both treat this as a temporary untrusted association.

Before starting an EAP conversation, the IoT device and the controller establish a CoAP level association, as required by CoAP-EAP [52]. These CoAP messages are necessary for exchanging nonces, which are later used together with the keys exported by EAP to derive cryptographic material for protecting the CoAP link between the device and the controller after bootstrapping. These initial CoAP exchanges provide the same functionality as the four-way handshake in Wi-Fi networks.

The IoT device initiates the bootstrapping process by sending a Non-confirmable (NON) Client Init CoAP request to the controller on the */boot* interface. The controller responds with a *Confirmable (CON) Controller Init* request. This request includes a 7-byte URI and a random nonce (nonce_c). The IoT device acknowledges the request with an *Ack init* message, which includes the same seven-byte URI and a random nonce (nonce_s) in the response.

After that, the EAP conversation begins. The CoAP messages exchanged during this conversation include EAP content as the payload. The controller initiates the conversation by sending an EAP identity request to the device, to which the device responds with its identity. In EAP-NOOB, all new devices use the same identity (noob@eap-noob.net). This informs the controller that the device will be bootstrapped with EAP-NOOB and that all EAP transactions for this device should be forwarded to the external AAA server over the RADIUS tunnel.

By receiving an unknown identity, the AAA server recognizes that a new device is requesting to join the network. It assigns the device a new identity and begins the Initial Exchange with an Elliptic Curve Diffie-Hellman (ECDH) exchange. The EAP-NOOB specification currently only supports ECDHE Curve25519. However, we decided use the NIST P-256 curve instead, since it is supported by hardware in the Firefly motes. The Initial Exchange always ends in an EAP-Failure message indicating that authentication is not yet successful.

Once the Initial Exchange is completed, the device starts blinking its LED light and waits for the user to transfer an OOB message to the AAA server. The EAP-NOOB specification defines a Waiting Exchange that the device can perform to check whether the OOB message has yet been delivered. However, it also specifies that configurable timers may be used to rate-limit the frequency of these exchanges. This allows for resource-constrained devices to limit the amount of requests made to the server. Similarly to the Initial Exchange, the Waiting Exchange also ends in an EAP-Failure.

After the OOB message has been delivered, the device and the AAA server finish the bootstrapping process with a Completion Exchange. This exchange verifies successful authentication and ends with the creation of a persistent association between the peer device and server. The AAA server also sends part of the MSK key to the controller. As explained earlier, the controller uses it together with the nonces from the initial CoAP packets to derive cryptographic key material. At this stage, the controller adds the new device to the list of authorized devices and finalizes it membership in the RPL network. If a new device is not authenticated within a reasonable time after initially joining the RPL network, the controller blacklists and then removes it from the network. The entire bootstrapping sequence is verified in a live real-world deployment [63].

## 8. Evaluation

In this section, we explain details of the design decisions and adaptations that we used for our implementation. Furthermore, we also analyze the code footprint of our implementation and the execution times of the different bootstrapping phases.

### 8.1. Optimizations

Because we rely on the stackless protothreads of Contiki-NG, we were forced to use static memory for storing the application state, rather than allocating memory on a per-message basis. We also made slight adjustments to the default Contiki-NG configuration to fit the entire peer implementation in memory. Most notably, we reduced the size of the IPv6 buffer to 500 bytes and increased the default stack size from 2 to 4 kB. We also had to disable certain low-power modes (modes 2 and 3) of the Firefly motes that restricted the amount of RAM to half of the total capacity (16 kB). Energy saving power mode 1 can still be used with our implementation.

We initially experimented with the rtimer library in order to implement the OOB step with the required precision. It is more accurate than the other timers of Contiki-NG and provides very reliable scheduling of real-time events. Using rtimers will naturally result in the highest throughput achievable on the OOB channel. However, we soon realized that the expiration of an rtimer causes a system interrupt, which interferes with any ongoing parallel EAP conversations. It is important to remember that EAP-NOOB requires the devices to output the OOB message while periodically executing a Waiting Exchange in parallel to check if the OOB message was delivered to the server. Because of these design requirements, we decided to use the etimer library instead. Replacing rtimers with less accurate etimers made our implementation simpler but slightly increased the time required for transferring OOB messages.

### 8.2. Evaluation of Code Footprint

**Figure 7:**
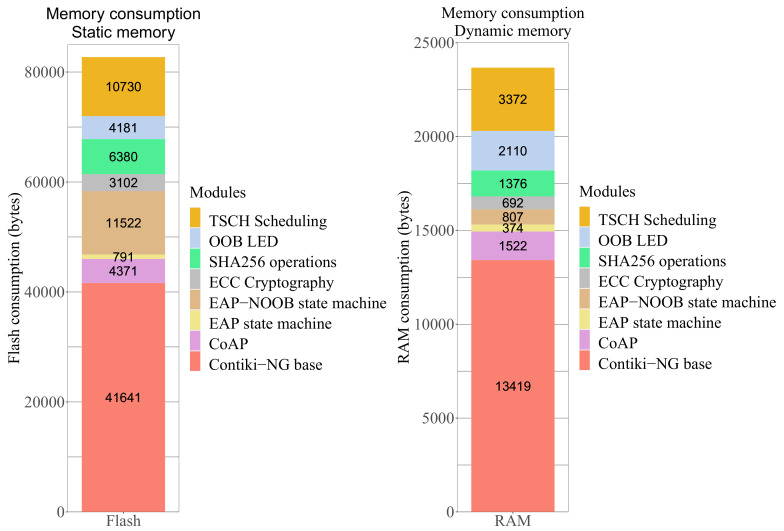
Memory consumption in Contiki. (**Left**) Static memory: Flash; (**Right**) Dynamic memory: RAM.

Our bootstrapping solution on the peer devices consumes approximately 69 kB of flash memory. The maximum RAM consumption of the device is approximately 20 kB. In Figure 7, we illustrate the sizes of each component of our bootstrapping solution. The left hand side shows the flash memory consumption and the right hand side shows the RAM consumption.

For detailed usage of each component, we extracted and divided the data into seven modules as follows: First, we use a basic implementation of Contiki-NG to get the memory requirements of the OS itself. These data are obtained by running the *Hello World* example. We then add the CoAP library with a basic example of receiving and sending messages. The CoAP module also includes the integrated UDP library. The evaluation of the remaining modules is performed over the Contiki-NG base plus the CoAP (over UDP) implementation.

The measurements for EAP-NOOB include all of its sub-components, such as its state machine and the JSON parsing functionality. However, it does not include the memory consumed by cryptographic operations. This information is separately illustrated in the ECC Cryptography and SHA256 modules. This is because we use the Public Key Hardware Accelerator (PKA) driver available on the Firefly motes for elliptic curve secp256r1 and SHA-256 hash operations. Keeping these data separate can also help in future work to investigate if these operations can be performed without additional hardware. OOB LED shows the memory requirements for the blinking LED light process.

Contiki-NG offers two MAC layer scheduling options: Carrier Sense Multiple Access (CSMA) and Time Slotted Channel Hopping (TSCH). It allows for communication with other motes with CSMA without any scheduling tool. In Figure 7, we indicate the overhead TSCH Scheduling when compared to the standard CSMA. It is evident that TSCH scheduling significantly increases the memory consumption and should only be used in critical networks that require high reliability and deterministic behavior.

### 8.3. Evaluation of the Message Size

**Figure 8:**
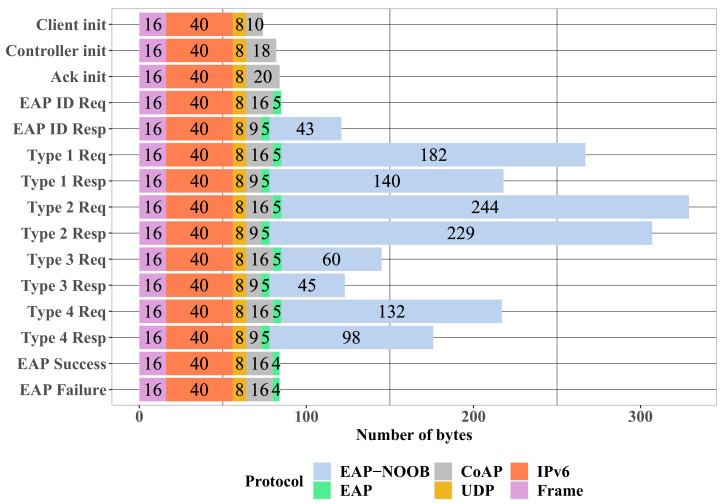
Message size of full nimble out-of-band authentication for Extensible Authentication Protocol (EAP-NOOB) exchange.

The size of each message is a crucial concern for resource-limited IoT devices. Whereas, for example, smart light bulbs are not constrained by the available power, many battery operated smart home and automation devices need to carefully consider the number of bytes sent and received over the radio interface. Figure 8 shows the number of bytes for each message type in our implementation. The overhead of each protocol layer is shown separately. The UDP, IPv6, MAC, and physical layers add a constant overhead of 64 bytes to all messages.

The messages that are necessary for bootstrapping a device with EAP-NOOB range from 74 to 329 bytes in size, which is arguably a significant amount of data, as can be seen in Figure 8. However, any protocol that provides strong security properties, such as perfect forward secrecy and cryptographic agility, without assuming any pre-configured credentials must at least exchange fresh public keys, nonces, and lists of supported cryptographic algorithms. Therefore, the sizes of the EAP-NOOB messages are justifiably large. Moreover, since the bootstrapping step is performed only once during the initial device setup, consuming additional resources for strong security is reasonable. Nevertheless, the overhead of EAP-NOOB could be further reduced by minor tweaks, such as using the more light-weight Concise Binary Object Representation (CBOR) [64] for messages instead of the JavaScript Object Notation (JSON) currently described in the specification. Finally, it is worth noting that the overhead of EAP itself is rather small, only 4–5 bytes per packet. Given this low overhead and the benefits of EAP discussed in this paper thus far, it becomes evident that using the EAP framework for device bootstrapping is a reasonable choice.

### 8.4. Evaluation of Time

**Figure 9:**
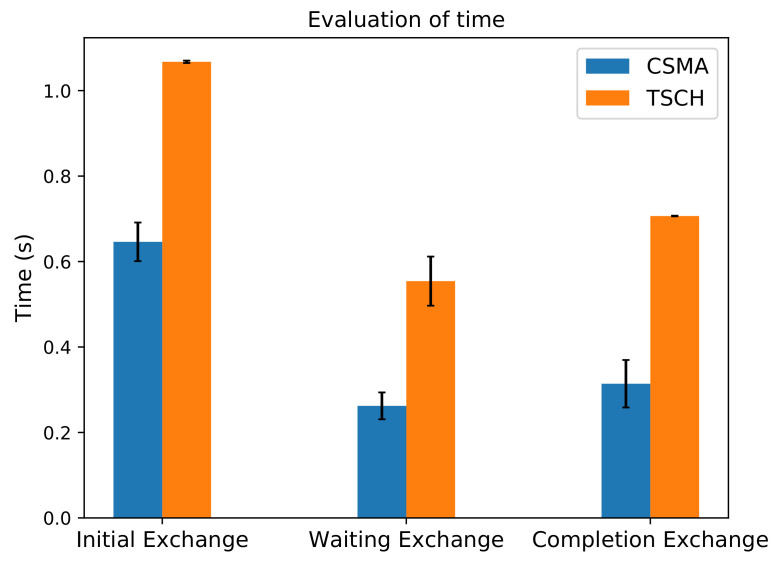
Time for each phase with Carrier Sense Multiple Access (CSMA) and Time Slotted Channel Hopping (TSCH).

Finally, we also measure the execution times needed for completing the required EAP-NOOB exchanges. We compare both of the previously presented MAC layer scheduling options (CSMA and TSCH). SMA scheduling outperforms TSCH scheduling, as is evident in Figure 9. This is expected, since our experiment setup does not take into account packet loss, interference, or bootstrapping over several hops. We plan to continue our evaluation with more complex network topologies in future.

## 9. Discussion

Network selection is one important aspect of our bootstrapping solution that we have not discussed thus far. A newly unboxed IoT device has no mechanism of knowing the appropriate network to which it should try to connect. In the presence of several networks, we require the device to perform the EAP-NOOB Initial Exchange with each of them and then transfer one URL per available network over the OOB channel. URLs are accompanied by network meta information obtained by the device. This meta information can include beacon payloads, which may help the user to identify his network. A similar approach to network selection is also used by Zigbee. Naturally, the OOB channel is limited by the number of bytes that can be transferred reliably in a few seconds. If there are more than 2–3 potential networks, a simple blinking LED light cannot transfer the OOB messages that are required for the EAP-NOOB protocol to successfully complete. Additional hints can also be used in order to reduce the number of potential networks. For example, Zigbee controllers include an “Allow Join” flag in their beacon when users are adding a new device. Controller devices also ensure that this flag is propagated to all routers in the network. A new device being added can examine this flag to limit the number of potential networks it probes.

One of the most serious threats against our implementation of EAP-NOOB is that we rely on HTTPS and a web browser on the user’s phone for delivering the OOB message between the device and the server. We expect users to identify the meta information of their network and the URL of their management server before entering any login credentials and adding a new device. Otherwise, an attacker can setup a network that mimics the honest user’s home network and a management server whose URL is only subtly different from what the user expects. Consequently, the attacker’s fake management server could capture either the honest user’s login credentials or take control of the new device. To avoid such attacks in practice, users must verify that the URL received over the OOB message indeed opens a secure HTTPS connection to the management server where he has an account. These attacks require the attacker’s network to be within range of the device being added and a careless user who does not check the URL before entering his login credentials while adding a new device. The usual phishing countermeasures apply here: user education and logging into the management server through a bookmark or a trusted portal page before scanning the blinking light. We also counter the chances of such an attack in our implementation. On the device side, we ignore server that send non HTTPS links.

Our Android application includes additional countermeasures to prevent users from inadvertently opening malicious URLs. We allow users to enter the details of their management server (URL and login details) before adding any device. Thus, instead of showing all the URLs received over the OOB channel to the user, we only show the one for the user’s management server. In practical real-world deployments, the user may be asked to download a companion smartphone application before bootstrapping an IoT device. The smartphone application might come pre-configured with the correct server URL. Such pre-configuration inherently protects careless users from registering their new devices with an attacker.

EAP-NOOB, like any EAP method, requires the setting up of a trust relation between the controller and management server. However, from then on, it saves the work of performing any additional configuration when new devices are added. This initial trust configuration requires setting up of peering between the controller device and AAA management server such that the EAP packets are securely forwarded. This peering is typically based on protocols, such as RADIUS or DIAMETER. While such a configuration is routine for small office and enterprise environments, most home users will find this trust configuration problematic and cumbersome. There are however several methods that can make this easier. First, controller devices can be pre-configured to connect to a commercial cloud-hosted AAA management server, possibly provided by the manufacturer of the controller device. Second, the routing to the user’s management server could be set up in the web console of the controller device. Although these methods for simplifying the peering process do not require any protocol changes, they do require new software on controller devices. A relatively new standard specification [65] even allows automated dynamic discovery of AAA servers. However, it is not yet widely-implemented or deployed. From this discussion, it is apparent that improving the usability of setting up trust relations (especially in home networks) requires further research.

One obvious question that still needs to be answered is who owns and runs the management server in the cloud infrastructure? Thanks to the EAP architecture, our proposed solution is flexible enough, such that the management server and the associated cloud service can be provided by the manufacturer of the new device being added, the manufacturer of the controller device, or any other commercial cloud-hosting services. It is possible that the cloud-based management servers will be provided by various service providers for a fee, which may be subsidized with advertising and premium features. Vendors of controller devices such as the Cozify Hub [66] could also provide this service to home users. Ultimately, the choice is dictated by the user: he needs (a) an account on the AAA server; and (b) setup the peering so that EAP packets can be correctly routed between the new device and his AAA server.

Thus far, we have discussed the benefits of EAP for bootstrapping IoT devices. We have also shown that lightweight standards–compliant implementations of EAP are indeed possible on resource-constrained devices. We specifically chose EAP-NOOB for our implementation, since it was the only EAP method that can work without requiring any manufacturer provisioned credentials. The usability of scanning blinking lights and setting up of the peering between the controller and the AAA management server are important aspects that require thorough further research.

## 10. Conclusions

We studied protocols for secure bootstrapping of resource-constrained smart home and automation devices. In our study, we looked at secure bootstrapping processes that are defined in standards specifications as well as currently available off-the-shelf devices. We observed that several standards, such as Z-Wave, now realize the need for identifying and configuring individual devices rather than relying on network-wide shared secrets. We then proposed a bootstrapping method that relies on a cloud-based AAA server and EAP. We verified the feasibility of EAP-NOOB as a bootstrapping mechanism by implementing it on resource-constrained devices with an embedded OS. Our implementation also developed a novel OOB channel that relies on a tiny LED bulb and can transfer data at approximately 80 bps, even on an embedded platform. Although secure bootstrapping is still an open research topic, our paper discusses important design principles that can help protocol engineers of forthcoming standards and devices.
